# Scalar and Ignorance Inferences Are Both Computed Immediately upon Encountering the Sentential Connective: The Online Processing of Sentences with Disjunction Using the Visual World Paradigm

**DOI:** 10.3389/fpsyg.2018.00061

**Published:** 2018-01-31

**Authors:** Likan Zhan

**Affiliations:** Institute for Speech Pathology and the Brain Science, School of Communication Science, Beijing Language and Culture University, Beijing, China

**Keywords:** scalar implicatures, ignorance inferences, disjunction, grammatical processes, visual-world paradigm

## Abstract

Accounts based on the pragmatic maxim of quantity make different predictions about the computation of scalar versus ignorance inferences. These different predictions are evaluated in two eye-tracking experiments using a visual world paradigm to assess the on-line computation of inferences. The test sentences contained disjunction phrases, which engender both kinds of inferences. The first experiment documented that both inferences are computed immediately upon encountering the disjunctive connective, at nearly identical temporal locations. The second experiment was designed to determine whether or not there exists an intermediate stage at which the truth of the corresponding conjunction phrase is ignored. No such stage was found.

## Introduction

An utterance in ordinary conversation often expresses information that is stronger than its literal meaning (Grice, 1975). Among such utterances are disjunctions such as [1a]. Literally, the disjunction [1a] is true when at least one of the two disjuncts [2a, 2b] is true. When the two disjuncts [2a, 2b] are both true, the corresponding conjunction [1b] is also true. In ordinary conversation, however, hearing the disjunction [1a] often makes the hearer infer that the corresponding conjunction [1b] is false (*scalar implicature*); and infer that the speaker doesn’t know whether either of the two disjuncts [2a, 2b] is true or false (*ignorance inference*). The two inferences result in that the disjunction’s actual interpretation is stronger than its literal meaning. It is widely accepted that the two inferences are both generated from a disjunction, but accounts differ in whether they are pragmatic or grammatical.

1. The two complex statements
     a. *John’s box contains a cow or a rooster*.
     b. *John’s box contains a cow and a rooster*.
2. The two disjuncts
     a. *John’s box contains a cow*.
     b. *John’s box contains a rooster*.

First, the pragmatic account contends that both inferences are derived from some post-compositional, pragmatic processes. This account was pioneered by Grice (1975). According to Grice (1975), a speaker sticking to the cooperative principle should be as informative as necessary (maxim of quantity) and should say only things he or she believes to be true (maxim of quality). Hence, a cooperative speaker should assert the strongest statement that he or she is in a position to make. Hearing the speaker’s assertion, a hearer then infers that the speaker was not in a position to assert any of the alternative statements that are stronger than the speaker’s assertion. The alternative statements constructed from the disjunction [1a] are comprised of two subsets: the first subset is constructed from the Horn scale (Horn, 1972), i.e., [1a, 1b], and the second subset is constructed from the constituents of the disjunction, i.e., [1a, 2a, 2b]. All the relevant alternatives [1b, 2a, 2b] generated from the disjunction are stronger than the disjunctive statement [1a]. Hence, upon hearing the disjunctive statement [1a], a hearer infers that the speaker was in a position to determine the disjunction [1a] is true (maxim of quality), but was not in a position to determine any of the three alternatives [1b, 2a, 2b] is true, otherwise the cooperative speaker would have used these alternative(s) (maxim of quantity). The hearer therefore must compute a *primary inference* that the speaker was not in a position to assert that the three alternatives [1b, 2a, 2b] are true. The hearer then needs to judge whether or not the speaker is likely to have an opinion about the truth of the alternatives; this is called the *competence assumption* (Sauerland, 2004; van Rooij and Schulz, 2004). If the hearer makes the competence assumption, he or she infers that the alternatives are false. This process gives rise to *scalar implicatures*. If the hearer does not make the competence assumption, he or she infers that the speaker is ignorant about the truth-value of the alternatives. This leads to an *ignorance inference*. To derive the two required inferences of the disjunction, one needs to hypothesize that the speaker is opinioned on the alternatives derived from the Horn scale, i.e., [1a, 1b], but is not opinioned on the alternatives derived from the constituents of the disjunction [1a, 2a, 2b], resulting in the *scalar implicature* denying [1b] and the *ignorance inference* relative to [2a, 2b].

Second, the hybrid account contends that scalar implicatures are derived using a compositional, grammatical process, whereas ignorance inferences are derived by a post-compositional pragmatic process. On the hybrid account, interpreting a statement begins with hearers determining whether or not they parse the speaker’s assertion using a phonologically null “exhaustification” operator (Fox, 2007). If the statement is parsed without this operator, the result is the literal meaning. If the statement is parsed with this operator, the result is an interpretation that includes scalar implicatures and strengthened meanings. Like the pragmatic account, the hybrid account begins the derivation of inferences by establishing a set of relevant alternatives that the speakers might have used in place of the assertion that the speaker made. The covert exhaustification operator is then applied to both the asserted statement and the relevant alternatives. The exhaustification operator is similar in meaning to the focus operator *only*. The output of the application of the exhaustification operator is a conjunction of propositions. One proposition is that the asserted statement is true. Another proposition is that all relevant alternatives that are not entailed by the asserted statement are false. According to the hybrid account, the exhaustification operator is part of the on-line composition of meaning, rather than post-compositional as on the pragmatic account. Hence, scalar implicatures should be observed on-line, at that point in sentence processing when the lexical item that triggers the exhaustification operator is encountered. On the hybrid account, this can happen when the lexical item is in the middle of a sentence, or at the end. In terms of the disjunction, a scalar implicature should be observed at that point in sentence processing when the sentential connective *or* is encountered, because the sentential connective *or* is the lexical item that triggers the exhaustification operation. With regards to the ignorance inference, by contrast, Fox (2007, 2014) maintains that this inference is derived from maxims of conversation, as on the pragmatic account. On the other hand, Chierchia (2004, 2017) contends that the ignorance inference results from the contradiction that is generated in computing the scalar implicature. As discussed earlier, the exhaustification operator that is triggered by disjunction [1a] can apply either to the Horn scales [1a, 1b], or to the domain alternatives [1a, 2a, 2b]. If the former, then no contradiction is generated; this yields the scalar implicature. If the latter, a contradiction is generated. In this case, the application of the exhaustification operator yields a meaning according to which the disjunction [1a] is true, and the two contradictory disjuncts [2a, 2b] are both false. When a contradiction is derived, the hearer arrives at the ignorance inference.

Third, the radically grammaticalized account put forward by Meyer (2013) contents that both inferences are derived inside the grammatical system of the language apparatus, rather than in the pragmatics. According to this account, an asserted proposition *S* always covertly attaches to an epistemic operator, *K*. Asserting the statement *S*, then, amounts to the assertion *K(S)*, i.e., the speaker knows or believes *S*. When a statement is parsed with the exhaustification operator, *EXH*, the exhaustification operator can apply either above or below the epistemic operator, leading to two legitimate readings, *EXH-K(S)*, *EXH-K-EXH(S)*. When the statement includes the disjunction connective, as in [1a], both readings give rise to the ignorance inference, based on the domain alternatives [1a, 2a, 2b]. However, the two readings yield different inferences when the alternatives include the Horn scales [1a, 1b]. The first reading results into a weaker inference (or primary inference) than the corresponding conjunction, i.e., the speaker is not in a position to know that the corresponding statement with conjunction [1b] is true, resulting in an ignorance inference relative to the conjunction [1b]. The second reading results in a scalar implicature, i.e., the speaker is in a position to know that the corresponding conjunction [1b] is false.

The different predictions made by the three accounts are summarized as **Table 1**. First, the pragmatic account regards the ignorance inference as being triggered by the conversational maxims, and as being the output of a domain general reasoning procedure. As it applies at the level of speech acts, the ignorance inference has to be post-compositionally processed and should not be observed until the offset of the test sentences (Sauerland, 2012). The scalar implicature emerges from the ignorance inference to the Horn scales (primary inference), together with the hearer’s competence assumption about the speaker. There should exist an intermediate step where participants are ignorant about the truth-value of the corresponding conjunction, and the scalar implicature should occur temporally later than that of the ignorance inference (Chemla and Singh, 2014a,b). Second, the hybrid account deems the scalar implicature as being triggered by a covert lexical operator. Because the output of a domain specific computation takes place within the linguistic system, the scalar implicature is compositional and could be observed prior to the offset of the test sentences. Researchers who advocate the hybrid account differ in the exact mechanisms of how the ignorance inference is derived (Fox, 2007, 2014; Chierchia, 2017), but the general view is that the ignorance inference is derived within the pragmatic component of the language apparatus. Because pragmatic processes normally follow grammatical processes, the ignorance inference is predicted to occur later than scalar implicatures (Chemla and Singh, 2014a,b). As scalar implicatures are derived in a single step, there should be no stage in processing at which an ignorance inference (or primary inference) is applied to the corresponding conjunction. Third, the radical grammatical account (Meyer, 2013) regards both inferences as being derived from the lexical compositional system. Both the scalar implicature and the ignorance inference should arise prior to the offset of the test sentences and should occur almost at the same time. The two supposed legitimate readings differ in whether the primary inference or ignorance inference on the corresponding conjunction should occur. The first reading *EXH-K(S)* ends up in a weak implicature where the truth-value of the corresponding conjunction is ignored. The second reading *EXH-K-EXH(S)* ends up in a scalar implicature where the corresponding conjunction is negated.

**Table 1:** Predictions made by different accounts.

Account	Scalar implicature vs. Ignorance inference	Primary inference
Pragmatic	Scalar implicature > Ignorance inference	Yes
Hybrid	Scalar implicature < Ignorance inference	No
Grammatic
EXH-K(S)	Scalar implicature = Ignorance inference	Yes
EXH-K-EXH(S)	Scalar implicature = Ignorance inference	No

To adjudicate between the three accounts (mainly the pragmatic account and the hybrid account), roughly two clusters of studies have been conducted in literature. The first cluster explored whether the scalar implicature is computed locally or globally. If it is a post-compositional operation at the level of speech acts, then the scalar implicature should only be globally computed and could not be locally computed. If it is a compositional lexical operation, then the scalar implicature should also possible be locally computed. There exist two ways to define global and local: whether the scalar implicature can be incrementally observed, i.e., prior to the offset of the test sentences; or whether the final comprehension of a complex statement is based on the scalar implicature applied on the main clause or applied on the subordinate clause. Using visual world paradigm, researchers have found that the scalar implicature can be incrementally processed, i.e., prior to the offset of the test audios (Breheny et al., 2006, 2013; Grodner et al., 2010; Degen and Tanenhaus, 2015; Foppolo and Marelli, 2017), although under certain experimental settings the processing could be delayed (Huang and Snedeker, 2009). Using complex statements where the scalar quantifiers are embedded under other words such as the universal quantifier *each*, researchers have found that both the global reading (Geurts and Pouscoulous, 2009) and the local reading (Clifton and Dube, 2010; Chemla and Spector, 2011; Chemla et al., 2017) are possible to be constructed. The first cluster of studies seems support the hybrid account. But the second cluster of studies give a different answer. The second cluster explored whether the scalar implicature is a domain specific, automatic process or a domain general, controlled process. If it is a compositional lexical operation, then the scalar implicature should be domain specific, and should be automatically triggered by the scalar quantifiers, regardless of other cognitive processes. The strengthened meaning should be the default meaning, even though it is more complex than the literal meaning. On the contrary, if it is a post-compositional operation at the level of speech acts, the scalar implicature should be a domain general process and should be constrained by other cognitive processes, such as memory. The strengthened meaning should be more difficult to access. Previous literature have found that both the participants’ epistemic status (Bergen and Grodner, 2012) and their available working memory sources (Marty and Chemla, 2013) affected the way a scalar implicature is computed. Furthermore, accessing the strengthened meaning is more time consuming (Bott and Noveck, 2004). So the second cluster of studies seem support the pragmatic account.

To summarize, previous studies mainly focused on the scalar implicature entailed by the existential quantifier *some*, i.e., *some but not all* (but see, Chevallier et al., 2008, on disjunctions). No clear-cut evidence so far has been observed to favor one account over another. Furthermore, the ignorance inference engendered by the disjunction has not been experimentally tested in literature.

To recap, accounts differ in the temporal sequences in which the scalar implicature and ignorance inference are computed online. Accounts also differ in whether or not a disjunction (temporally or permanently) triggers an ignorance inference to the corresponding conjunctive statement. To explore these two questions, we reported two eye-tracking studies using the visual world paradigm (Cooper, 1974; Tanenhaus et al., 1995; Zhan et al., 2015). Experiment 1 explored the temporal sequences of the scalar implicature and ignorance inference. Experiment 2 explored the problem of the ignorance inference applied to the corresponding conjunction.

## Experiment 1

### Method

#### Participants

Thirty-seven postgraduate students from the Beijing Language and Culture University participated in this experiment. All the participants were native speakers of Mandarin Chinese, with normal or corrected normal visions. They were paid 30CNY (approximately $5) for their participation.

#### Stimuli

**FIGURE 1:**
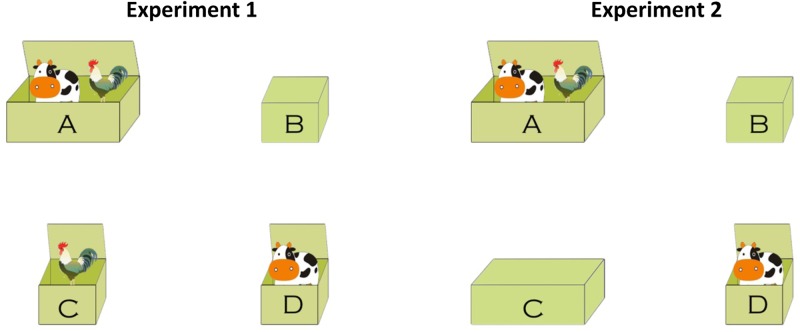
An example of the test images used in Experiment 1 (left) and Experiment 2 (right).

A test image involved two animals and four boxes situated at the four quadrants (**Figure 1**). Two properties of the boxes were manipulated: the size and the closeness of a box. The size of a box influenced the animals included in the box, but not participants’ epistemic status on that box. A big box always contained two different animals, while a small box always and only contained one animal, no matter whether the box was closed or not. The closeness of a box influenced participants’ epistemic status on that box, but not the animals contained in that box. If a box was open, both the speaker and the hearer were in a position to know what animal(s) were contained in that box. If a box was closed, both the speaker and the hearer were not in a position to know what animal(s) were contained in that box.

The test image in Experiment 1 (**Figure 1**, left) consisted of one big box and three small boxes. In the given example, the two animals were a cow and a rooster. The big box *A* was open and contained both a cow and a rooster. Two of the three small boxes *C* and *D* were also open and contained a rooster and a cow, respectively. The third small box *B* was closed. Henceforth, participants were unable to know which animal was in box *B*. But the size of box *B* is small, so participants knew that the small box *B* contained only one animal: it was either a cow or a rooster, but not both. Sixty images like the left panel of **Figure 1** were constructed, with the spatial locations of the four boxes being counterbalanced and with the two involved animals also being changed across images.

**FIGURE 2:**
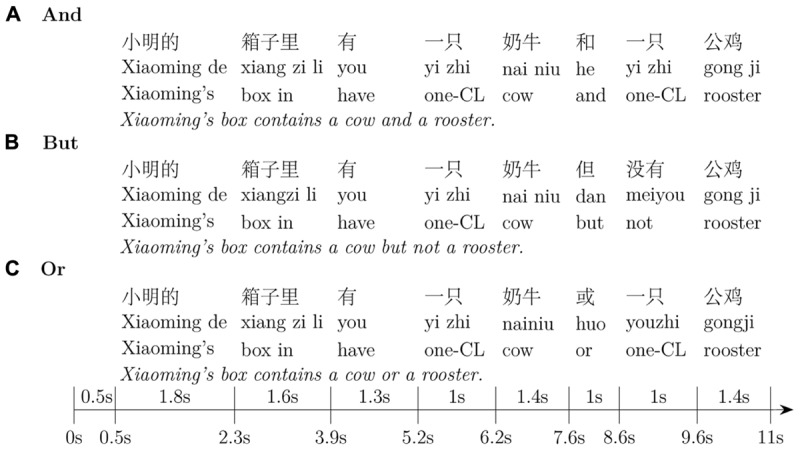
An example of the test sentences used in Experiment 1 and Experiment 2.

Three test sentences (**Figure 2**) were constructed to each test image: A conjunctive statement (**Figure 2A**), a *but*-statement (**Figure 2B**), and a disjunctive statement (**Figure 2C**). One more statement in the form of *Xiaoming’s box doesn’t contain a rooster but a cow* was used as a filler and was not analyzed in our studies. In each statement, one animal such as the cow in our example was mentioned as the object of the first proposition, while the other such as the rooster was mentioned as the object of the second proposition, respectively. Participants were told that one of the four boxes belongs to *XiaoMing.* Participants’ task was to find XiaoMing’s box according to the test sentence they heard, and press the corresponding button. Participants’ online eye-movements on the four boxes as they were listening to the test audios, as well as the boxes participants behaviorally chose, were recorded and used to deduce how the scalar implicature and ignorance inference were processed. The 240 test sentences were then divided into four groups, with each group containing 15 conjunctions, 15 disjunctions, 15 *but*-statements, and 15 filler statements. Each participant saw all the 60 images and heard only one group of the test audios.

The test sentences were recorded by a female native speaker of Mandarin. To make them the same in length and consistent in intonation, all the test audios were exactly the same except for the objects of the two merged disjuncts/conjuncts. To achieve this, we first recorded four example statements, such as (A–C) in **Figure 2**, as well as all the objects that were going to be used in our studies, such as *pig* and *horse*. We then replaced the two objects in the example statements, i.e., *cow* and *rooster*, with each pair of the recorded objects, such as *pig* and *horse*, resulting in the full list of our test audios. We did a pilot test by asking several native Mandarin-speakers to judge the naturalness of the test sentences in Mandarin, all the interviewees judged the test sentences to be natural Mandarin sentences. The length of the test audios is marked on **Figure 2**.

Given our experimental design, all boxes would be possible candidates, unless a box was ruled out by the computed inference. First, if the ignorance inference to the domain alternatives is engendered from the disjunctive statement [1a], then participants should be in a position not to know the truth values of the two domain alternatives [2a, 2b]. All situations where participants know the truth of the domain alternatives will be ruled out. A small open box means that participants know the truth value of a corresponding domain alternative. Henceforth, computing the ignorance inference will rule out all the small open boxes and result in significant fewer fixations on these boxes. Second, if the scalar implicature and the Horn scales are derived from the disjunctive statement [1a], then participants should be in a position to know the corresponding conjunctive statement [1b] is false. All the situations where the participants are not in a situation to know the corresponding conjunctive statement [1b] is false will be ruled out. In this case, the excluded situations consist of not only the situations where the speaker knows that the corresponding conjunctive statement [1b] is true, but also the situations where the speaker is ignorant about the truth of corresponding conjunctive statement [1b]. In our experimental design, a big open box means that participants know the conjunction [1b] is true. A big closed box means that participants don’t know whether the conjunction [1b] is true or false. Computing the scalar implicature will rule out all the big boxes and lead to significant few fixations on these boxes, regardless of whether they are open or closed. Third, if the inference derived from the Horn scales is an ignorance inference but not a scalar implicature, then participants will not derive the inference that the conjunction [1b] is false. In this case, only the situations where participants know that the corresponding conjunction [1b] is true will be ruled out, not the situations where participants don’t know the truth of the conjunctive statement. This so-called weak inference will rule out the big open boxes, but not the big closed ones, resulting in significant fewer fixations only on the big open boxes.

#### Procedure

Participants were seated approximately 64 cm from a 21 inch, 4:3 color monitor with 1,024^∗^768 pixel resolution. Twenty-seven pixels equaled approximately to 1° of visual angle. The sampling rate of the Eyelink 1000plus eye-tracker was 500 Hz. Viewing was binocular, but only the participant’s dominant eye was tracked. The auditory stimuli were presented via a pair of external speakers situated to the two sides of the monitor. At the beginning of the experiment, participants first saw an introduction of the experiment in Mandarin on the screen. The instruction briefly explained the experimental procedure as we described below.

**FIGURE 3:**
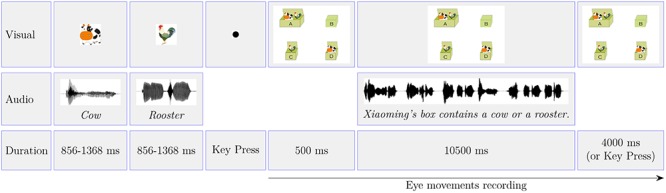
The time line of a typical trial used in Experiment 1. The time line of Experiment 2 is exactly the same as Experiment 1 except for the test image (**Figure 1**). The audios illustrated in the image are the English translations of the Mandarin Chinese used in the experiments.

After participants were comfortable with the experimental aim and the procedure, the experimenter then helped participants to perform the standard Eyelink calibration and validation routines. Each trial involved two animals. The time line of a typical trial is summarized in **Figure 3**. Participants first saw two images of one animal each printed on the screen in turn, along with the name of the animals played via the two loudspeakers situated at both sides of the screen. A black dot was then presented at the center of the screen. The participant was instructed to press the SPACE key while fixating on the dot. The press brought up the test image. The 500 ms after the onset of the test image, the test sentence began to play. The 4,000 ms after the offset of the test audio or pressing a key brought out a new trial. Participants’ task was to determine which box the test sentence was talking about and pressed the corresponding key on the keyboard as soon as possible. Participants’ eye movements were recorded from the onset of the test image to the offset of the trial.

### Results

#### Behavioral Responses

The correct response to a conjunction was the big open box containing the tokens of both conjuncts. The correct response to a *but*-statement was the small open box containing the token of the first conjunct but not the second conjunct. Participants’ behavioral responses to the disjunction, however, depended on whether the two inferences were processed. If participants computed neither the scalar implicature nor the ignorance inference, then all the four boxes in **Figure 1** were eligible selections. If the scalar implicature but not the ignorance inference was computed, then the big-open box *A* would be ruled out, and the remaining three boxes *B*, *C*, and *D* were eligible options. If the ignorance inference but not the scalar implicature was computed, then the two small open boxes *C* and *D* would be ruled out, and boxes *A* and *B* would remain to be eligible options. And the small-closed box B would not be chosen unless both the scalar implicature and the ignorance inference were computed.

**FIGURE 4:**
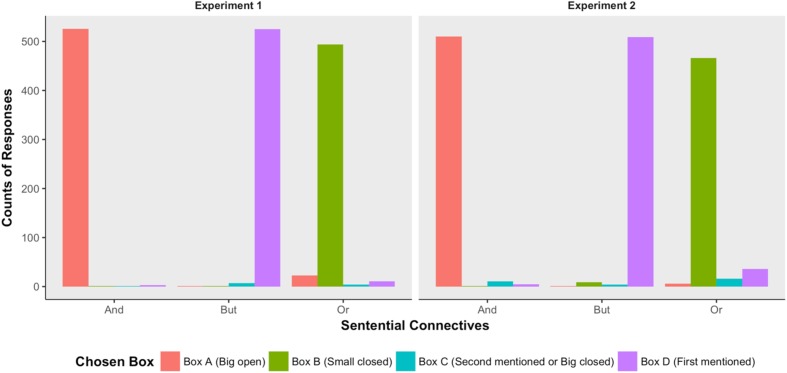
Participants’ behavioral responses observed in Experiment 1 (left) and Experiment 2 (right). To make the four boxes more meaningful, I labeled box A as “*Box A (Big Open)*,” and box B as “Box B (small closed).” The contents of Box C differ between the two experiments. In Experiment 1, box C was a small open box containing the animal that was mentioned in the second proposition; in Experiment 2, box C was a big closed box. So I labeled box C as “*Box C (Second Mentioned or Big Closed).*” Box D contained an animal that was mentioned in the first proposition (**Figure 2**), so I labeled box D as “*Box D (First Mentioned).*”

The left panel of **Figure 4** summarized participants’ behavioral responses in Experiment 1. In our experimental design, the correct answers to the conjunctions were the big open-boxes (Box A), and the correct answers to the *but*-statements were the small-open boxes (Box D) containing the animals that were mentioned in the first conjuncts. Things became complex when the test sentences were disjunctions. In our experimental setting, all the boxes were compatible with the literal meaning of the uttered disjunctions, so participants’ behavioral responses to the conjunctions could not be categorized as correct or incorrect. However, the boxes participants actually chose could inform us as to whether they computed the two proposed inferences or not. If participants computed the scalar implicature but not the ignorance inference, then they would choose boxes B, C or D. If participants computed the ignorance inference, then they would choose boxes A or B. If box B was the only choice participants made, it would suggest that participants computed both the scalar implicature and the ignorance inference. As we saw in the left panel of **Figure 3**, participants predominantly chose the big-open boxes, the first-mentioned small box, and the small-closed box, when they heard conjunctions, but-statements, and disjunctions, respectively. These findings suggested that both the scalar implicature and the ignorance inference were computed, and their computation was no later than the temporal location when participants overtly gave their behavioral responses.

However, the behavioral responses didn’t tell us when the two inferences occurred while participants were listening to the test audios. To explore how the test statements were processed online and when the two inferences occurred, we then analyzed participants’ eye movements on the test images as they were listening to the auditorily presented test statements.

#### Eye-Tracking Results

The test audios were 11 s long, and the eye-tracker had a sample rate of 500 Hz, so we had 5,500 sample points per testing trial. To process the eye-tracking data, we first deleted the samples where participants’ eye movements were not caught, such as when they blinked their eyes. This process roughly affected 10% of the recorded data. We then defined four equal-sized areas of interest in the test image, containing the four boxes, respectively. Third, we then coded the recorded data as follows: for a specific area of interest, the samples where participants’ fixations locating in that area was coded as 1, and the samples where participants’ fixations locating out of that area was coded as 0.

**FIGURE 5:**
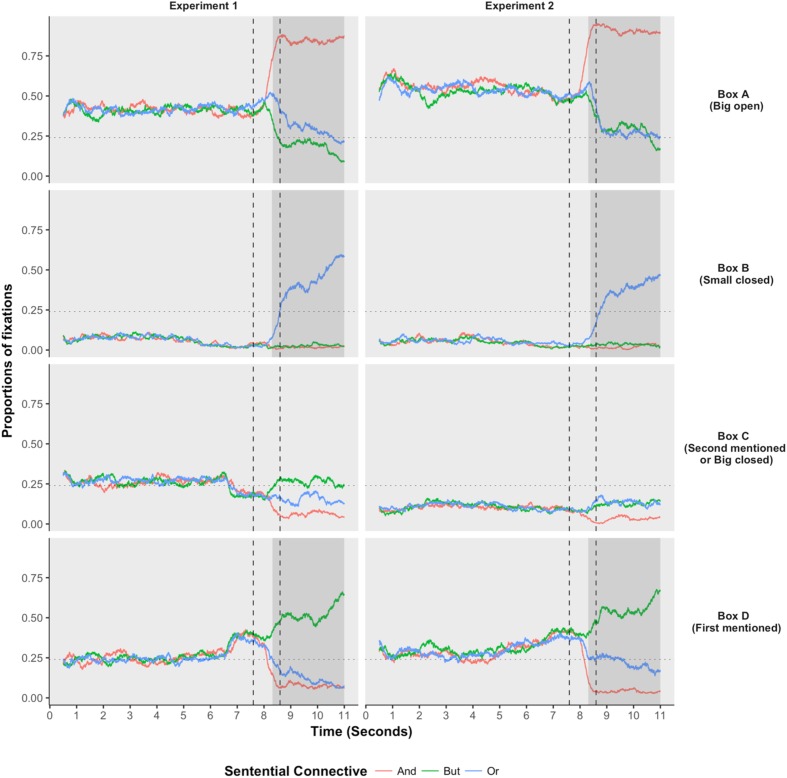
Participants’ eye-movements recorded in Experiment 1 (left) and Experiment 2 (right). The two vertical lines signified the onset and offset of the sentential connectives. The gray area means that a significant difference existed between the disjunction and the baseline condition (*p* < 0.05, Bonferroni adjusted). The baseline was the conjunction when the interested areas were Boxes A and B, and the baseline was the *but*-statement when the interest areas were Box D.

The results of Experiment 1 were summarized in the left panel of **Figure 5**, where the *x*-axis was the sample point where the eye movement was recorded and *y*-axis was the proportion of samples where participants located their eye fixations on a specific area of interest. The four panels depicted participants’ fixation patterns on the four areas of interest. The red, green, and blue lines illustrated participants’ fixation patterns when the test statements were conjunctions, but-statements, and disjunctions, respectively. We were interested in how participants’ fixation patterns were distributed among the four interest areas and how these distributions were influenced by hearing auditorily presented test sentences. The two dashed vertical lines illustrated the onset and offset of the sentential connectives. The horizontal dotted line labeled the proportion of 25%, illustrating the chance level of participants’ fixation patterns. A preferred box should be fixated at more than 25% of the recorded samples.

To conduct the statistical analysis, we fitted a binomial generalized linear mixed model (GLMM) to the data at each interest area of each sample point. The GLMM model contained one fixed term: *sentential connectives*. The baseline of this fixed items differed when the analyzed interest area, i.e., the analyzed boxes, were different. To be specific, the conjunction was chosen as the baseline when analyzing the big-open box, the disjunction was chosen as the baseline when analyzing the small-closed box, and the *but*-statement was chosen as the baseline when analyzing the first-mentioned box (Box D). To summarize, the sentential connective whose expected response was the same as the analyzed interest area would be chosen as the baseline. The GLMM model included two random terms: *participants* and *items*. The formula evaluated to the two random terms included both the intercepts and the slope of the *sentential connectives*. The model fitting process was conducted via *glmer* function from *lme4* package (Bates et al., 2015) under the *R* environment (R Core Team, 2016). The *p*-values obtained using Wald *z*-tests were then Bonferroni adjusted. The gray areas in **Figure 4** signified the temporal periods where a significant difference existing between the baseline condition and the disjunctive condition (*p* < 0.05, Bonferroni adjusted). The statistical results with the wrong answered trials being excluded from the analyses were the same as the statistical results with all trials being included, so we only reported the results with all trials being included in the analyses.

As we saw in the left panel of **Figure 5**, prior to the onset of the sentential connectives, no difference was observed between the three conditions, among all the four interest areas. These results provided reasonable bases that all the observed differences among the three conditions after the onset of the sentential connectives resulted from the sentential connectives, but not from other confounding factors. After the onset and prior to the offset of the sentential connectives, the disjunctive connective *or* had already triggered significantly different eye-movements than the baseline conditions among the three interest areas. And the starting points where a significant effect was first observed were summarized in **Table 2**. First, the disjunctive connective *or* had triggered significantly fewer fixations on the big-open box (Box A) than the conjunctive connective *and*; and this effect happened no later than 0.724 s after the onset of the sentential connectives. This suggested that the scalar implicature had already been fully processed prior to the offset of the sentential connectives. Second, the disjunctive connective *or* had also triggered significantly fewer fixations on the first-mentioned box (Box D) than the sentential connective *but*; and this effect happened no later than 0.714 s after the onset of the sentential connectives. This suggested that the ignorance inference had already been fully processed prior to the offset of the sentential connectives. Third, the disjunctive connective *or* had already triggered significantly more fixations on the small-closed box (Box B) than the sentential connectives *and* and *but*; and this effect happened no later than 0.690 s after the onset of the sentential connectives. Fourth, participants’ fixations on the second-mentioned box (Box C) were never bigger than the chance level (0.25), suggesting that the second-mentioned box was never regarded as a legitimate option to our test audios. These results confirmed the previous two observations that both the scalar implicature and ignorance inference have already been fully processed prior to the offset of the sentential connectives.

**Table 2:** The temporal locations where significant differences started to be observed.

Experiment	Interest area	Baseline	Trial onset	Connective onset
One	Big open (Box A)	And	8.324	0.724
	Small closed (Box B)	And	8.290	0.690
	First mentioned (Box D)	But	8.314	0.714
Two	Big open (Box A)	And	8.304	0.704
	Small closed (Box B)	And	8.388	0.788
	First mentioned (Box D)	But	8.310	0.710

*“Trial onset” illustrated the temporal locations based on the onset of the trial, i.e., the onset of the test image. “Connective onset” illustrated the temporal locations based on the onset of the sentential connectives*.

### Discussion

To summarize, Experiment 1 found that the scalar implicature and the ignorance inference were both locally computed. The effects triggered by the two inferences emerged no later than the offset of the sentential connectives. The two inferences occurred almost at the same time when participants were listening to the test audios. These findings suggested that the radical grammatical account put forward by Meyer (2013) was more reasonable than the pragmatic account (Horn, 1972; Fauconnier, 1975; Sauerland, 2004; van Rooij and Schulz, 2004; Russell, 2006; Spector, 2007; Geurts, 2009; Franke, 2011) and hybrid account (Chierchia, 2004, 2017; Fox, 2007, 2014; Fox and Hackl, 2007; Magri, 2009, 2011; Chierchia et al., 2012) in terms of explaining our data.

However, the big open boxes in Experiment 1 were able to be ruled out both by the scalar implicature and by the ignorance inference that applied to the corresponding conjunctions. Experiment 1 could not be used to determine whether a weak inference (or ignorance inference) had been (temporarily or permanently) computed, because this experiment didn’t contain a situation where the speaker was ignorant about the truth of the corresponding conjunction. To solve this problem, Experiment 2 introduced a big-closed box, where the speaker and the hearer didn’t know whether the corresponding conjunction was true or false. Under this experimental setting, if the computed inference to the corresponding conjunction is (temporarily or permanently) an ignorance inference, then the big open boxes, but not the big closed boxes would be ruled out. This would result in significant fewer fixations on the big open boxes, but not on the big closed boxes. In contrast, if the computed inference to the corresponding conjunction is a scalar implicature, then both the big open boxes and the big closed boxes would be ruled out. This would give rise to significantly fewer fixations on all big boxes, irrespective of whether they were open or closed. In Experiment 2, participants’ fixations on the big closed boxes will be crucial to differentiate between the two possibilities.

## Experiment 2

### Method

#### Participants, Stimuli, and Procedure

Thirty-six postgraduate students from the Beijing Language and Culture University participated in this experiment. All the participants were native speakers of Mandarin Chinese, with normal or corrected to normal visions. None of these participants had participated in Experiment 1. They were paid 30CNY (approximately $5) for their participation.

The stimuli and experimental procedure used in Experiment 2 were exactly the same as Experiment 1 with the following exception. In Experiment 1, a test image consisted of three small boxes and one big box. Two small boxes were open and contained two animals that were mentioned in the first and second merged propositions, respectively. In our example as illustrated in the left panel of **Figure 1**, the two-small open boxes were *C* and *D*, containing “cow” and “rooster,” respectively. In Experiment 2, however, the small box containing the animal that was mentioned in the second merged proposition was replaced by a big closed box. In our example, the second mentioned animal “rooster” was contained in box C. In Experiment 2, the small open box C was replaced by a big closed one, yielding the right panel of **Figure 1**.

### Results and Discussion

Participants’ behavioral responses in Experiment 2 (right panel of **Figure 4**) were similar to that observed in Experiment 1, indicating that replacing a small open box with a big closed box did not have a significant effect on participants’ behavioral responses.

Participants’ fixations patterns (right panel of **Figure 5**) and the onsets of the significant difference (**Table 2**) observed in Experiment 2 were also similar to that observed in Experiment 1. Furthermore, the big-closed box (Box C) always received fewer fixations than the chance level, regardless of the temporal positions and the sentential connectives, suggesting that a big box was never regarded as a valid option of the disjunction, regardless of whether the big box was open or closed.

## General Discussion

First, our results have important implications for adjudicating between different accounts of scalar implicatures and ignorance inferences. The pragmatic account (Horn, 1972; Fauconnier, 1975; Levinson, 2000; Sauerland, 2004; Russell, 2006; Schulz and van Rooij, 2006; Spector, 2007; Geurts, 2009, 2010; Franke, 2011) regards both inferences as pragmatic processes. A pragmatic process applies over speech acts while a speech act is derived from the whole statement, and as pragmatic processes, the two inferences are not expected to occur until the offsets of the test sentences (but see, Chemla and Singh, 2014a,b, for different viewpoints). Furthermore, the pragmatic account predicts that the ignorance inference should occur earlier than the scalar implicature (Chemla and Singh, 2014a,b). Our results are contradictory to their predictions. We observed that both the scalar implicature and the ignorance inference were computed prior to the offset of the sentential connectives *or* that triggered the two inferences. These two inferences occurred almost at the same time. These results are also contradictory to the hybrid account (Chierchia, 2004, 2017; Fox, 2007, 2014; Fox and Hackl, 2007; Magri, 2009, 2011; Chierchia et al., 2012), which regards the scalar implicature as a grammatical process, but regards the ignorance inference as a pragmatic process. A grammatical process should occur earlier than that of a pragmatic process. According to the hybrid theory, the scalar implicature should be locally computed, but the ignorance inference should not. The scalar implicature is expected to be processed earlier than that of the ignorance inference. Our results are in a par with the radically grammatical account (Meyer, 2013), which regards both the scalar implicature and the ignorance inference as grammatical processes. The two grammatical inferences are triggered by the same lexical item, i.e., the disjunctive connective *or* in our experiments, so the two inferences are expected to occur at the same time.

Second, Experiment 2 explored whether or not there is an intermediate stage between the literal meaning and the scalar implicature. This stage is called the primary inference by the pragmatic account. The findings showed that there is no such intermediate stage. These findings are contradictory to the pragmatic account (Horn, 1972; Fauconnier, 1975; Levinson, 2000; Sauerland, 2004; van Rooij and Schulz, 2004; Russell, 2006; Schulz and van Rooij, 2006; Spector, 2007; Geurts, 2009, 2010; Franke, 2011), but are consistent with both the hybrid account (Chierchia, 2004, 2017; Fox, 2007, 2014; Fox and Hackl, 2007; Magri, 2009, 2011; Chierchia et al., 2012) and the second reading of the radically grammatical account (Meyer, 2013) that the scalar implicature is not derived the maxims of conversations.

Third, there exist several upper-bounded construals that engender scalar implicatures, including the disjunctive connective *or* explored here and the existential quantifier *some* explored extensively in literature. The scales engendered by different scalar expressions are traditionally regarded as having the same properties. Recently, researchers have begun to realize that there might be a heterogeneity between different scalar scales (Doran et al., 2009, 2012; van Tiel et al., 2016). For example, the distinctness of the scalemates have been found to affect participants behavioral responses (van Tiel et al., 2016). Our experiments found that the disjunctive connective *or* induces both a scalar implicature to its Horn scales and an ignorance inference to its two disjuncts, which is different from other scalar expressions. Any robust theory of quantity-based implicatures should encompass the variety between different scalar expressions. Regardless of the varieties, these scalar expressions are not necessarily different from each other on the pragmatic-grammatical dimension. And the distinction on this dimension is crucial in differentiating different accounts. Previous studies (Breheny et al., 2006, 2013; Grodner et al., 2010; Degen and Tanenhaus, 2015; Foppolo and Marelli, 2017) suggest that the scalar implicature computed from the quantifier *some* is a semantic process. The two experiments I reported here also supported the idea that both the scalar implicature and the ignorance inference engendered from the disjunctive connective *or* are semantic processes. Furthermore, our preliminary data exploring the online processing of the modal verbs [*might*, *must*] and of the quantificational adverbs [*sometimes*, *always*] suggest that these scales also are immediately constructed once the modal verbs and the quantificational adverbs are encountered. Taken together, the available online processing data using the visual world paradigm tend to support the idea that the scalar implicature in general is a grammatical process.

## Ethics Statement

This study was carried out in accordance with the recommendations of Beijing Language and Culture University Committee on Human Research Protection with written informed consent from all subjects. All subjects gave written informed consent in accordance with the Declaration of Helsinki. The protocol was approved by the Beijing Language and Culture University Committee on Human Research Protection.

## Author Contributions

LZ designed, conducted the experiments, analyzed the data, and wrote the paper.

## Conflict of Interest Statement

The author declares that the research was conducted in the absence of any commercial or financial relationships that could be construed as a potential conflict of interest.
